# Recommended rates of azoxystrobin and tebuconazole seem to be environmentally safe but ineffective against target fungi

**DOI:** 10.1007/s10646-023-02619-w

**Published:** 2023-01-18

**Authors:** Libânia Queirós, Nuno Aguiar, Patrícia Pereira, Fernando J. M. Gonçalves, Artur Alves, Joana Luísa Pereira

**Affiliations:** 1 Department of Biology, University of Aveiro, Aveiro, Portugal; 2 CESAM (Centre for Environmental and Marine Studies), University of Aveiro, Aveiro, Portugal

**Keywords:** Azoxystrobin, Tebuconazole, Mixture toxicity, *Pyrenophora teres*, *Rynchosporium secalis*

## Abstract

The use of fungicides in agriculture has been playing a role in the enhancement of agricultural yields through the control of pathogens causing serious diseases in crops. Still, adverse environmental and human health effects resulting from its application have been reported. In this study, the possibility of readjusting the formulation of a commercial product combining azoxystrobin and tebuconazole (active ingredients – AIs; Custodia®) towards environmentally safer alternative(s) was investigated. Specifically, the sensitivity of non-target aquatic communities to each AI was first evaluated by applying the Species Sensitivity Distributions (SSDs) approach. Then, mixtures of these AIs were tested in a non-target organism (*Raphidocelis subcapitata*) denoting sensitivity to both AIs as assessed from SSDs. The resulting data supported the design of the last stage of this study, where mixtures of those AIs at equivalent *vs*. alternative ratios and rates as in the commercial formulation were tested against two target fungal species: *Pyrenophora teres* CBS 123929 and *Rhynchosporium secalis* CBS 110524. The comparison between the sensitivity of non-target aquatic species and the corresponding efficacy towards target fungi revealed that currently applied mixture and rates of these AIs are generally environmentally safe (antagonistic interaction; concentrations below the EC_1_ for *R. subcapitata* and generally below the HC_5_ for aquatic non-target communities), but ineffective against target organisms (maximum levels of inhibition of 70 and 50% in *P. teres* CBS 123929 and *R. secalis* CBS 110524, respectively). Results additionally suggest a potentiation of the effects of the AIs by the other formulants added to the commercial product at tested rates. Overall, this study corroborates that commercial products can be optimized during design stages based on a systematic ecotoxicological testing for ingredient interactions and actual efficacy against targets. This could be a valuable pathway to reduce environmental contamination during transition to a more sustainable agricultural production.

## Introduction

The negative impacts of the use of fungicides and other pesticides on human health and environmental quality are undeniable. Adverse effects provoked by these environmental contaminants in non-target aquatic and terrestrial communities, as well as in agricultural workers, have been reported (as reviewed in Rani et al. 2021). However, the use of these products has also several advantages such as the enhancement of agricultural yields through the control of pathogens causing serious diseases in crops.

Pesticide formulations are composed of active ingredients (AIs) and co-formulants that are added to the formulation for diverse purposes, like the improvement of stability and target delivery of the main components (*e.g.* Knowles 2008). In the EU context, the AIs are approved at the EU level, but the final commercial products are approved at the Member State/national level (Robinson et al. 2020), and not all Member States test them (OECD 2010). Notwithstanding, studies have shown that co-formulants can change the overall product toxicity (*e.g*. Nagy et al. 2020) and, in some cases, they can be even more toxic than the AIs (*e.g*. the adjuvant polyethoxylated tallowamine POE-15 was more toxic to human cells than the AI glyphosate; Mesnage et al. 2013). Overall, literature has been revealing that approved pesticides, including commercial formulation forms, can cause harm to non-target species (*e.g*. Duke 2018; Mesnage et al. 2015). Furthermore, these effects can be caused at concentration levels even below or within the range of regulatory thresholds (*e.g*. Mesnage et al. 2015). This suggests either inadequate testing of these products during design stages or a failure in the approval process. In previous studies, we found that two commercial formulations could be optimized for improved environmental compatibility. The mixture of AIs used in a commercial herbicide (*i.e*. terbuthylazine and nicosulfuron) caused antagonistic effects in both a non-target aquatic macrophyte (*Lemna minor*) and a target weed (*Portulaca oleracea*; Queirós et al., 2018). Furthermore, terbuthylazine dosed alone at 10-fold lower rates than commercially recommended was effective against the target weed. More recently, another commercial herbicide combining terbuthylazine with bentazone was tested for environmental safety considering sensitive aquatic (*Raphidocelis subcapitata*) and terrestrial (*Brassica napus*) organisms and effectiveness in the control of the target weed (*Portulaca oleracea*; Queirós et al. 2022). While recommended rates seemed to represent a remarkable potential risk to soil and also aquatic ecosystems, application rates 10-fold lower than recommended were effective in the control of the weed. Moreover, a one-way formulation including only bentazone, which is already available on the market, seems environmentally safer than the two-way formulation. In this context, we reasonably argue that a rethinking of available commercial formulations could be a feasible pathway to reduce the contamination and subsequent impacts of pesticides during the currently prioritized transition to a more sustainable agricultural production as advocated *e.g*. by the EU Green Deal. This rethinking would consist in a proper assessment of the mixture interactions among the different ingredients of the formulations and consequent effects to both non-target and target species. Concurrently, this assessment would allow to determine minimum rates needed to control the pest/weeds while ensuring the maximum protection to sensitive non-target species. In the present study, we capitalized on this reasoning and tested it using a commercial fungicide, Custodia®.

Custodia® (Adama® 2021a) is a commercial concentrated suspension with fungicidal action that is composed of the AIs azoxystrobin (120 g L^−1^) and tebuconazole (200 g L^−1^), along with other co-formulants (1,2-Benzisothiazol-3(2H)-one, sodium hydroxide, propane-1,2-diol and glyoxal). This broad-spectrum product, as well as other equivalents available on the market (*e.g*. Adama® 2021b), are indicated for application in diverse crops to treat powdery mildew and winter cereal diseases. Azoxystrobin and tebuconazole are combined in these formulations to control, for instance, the fungal species *Pyrenophora teres* CBS 123929 and *Rhynchosporium secalis* CBS 110524 that cause net blotch and scald or leaf blotch diseases, respectively, in barley (*e.g*. Fountaine et al. 2010; Liu and Friesen 2010). The recommended application doses of Custodia® in barley and other winter cereals range within 1–1.25 L ha^−1^, which translates into 120–150 g of azoxystrobin and 200–250 g of tebuconazole *per* ha (Adama® 2021a). In grapes, lower rates corresponding to 0.75 L of Custodia® ha^−1^ are recommended (equivalent to 90 g azoxystrobin and 150 g tebuconazole *per* ha) to control powdery mildew. Azoxystrobin is a synthetic strobilurin that inhibits mitochondrial respiration at complex III (cytochrome bc1 complex), by blocking the transference of electrons from cytochrome b to cytochrome c1 (NCB National Center for Biotechnology 2021). It is a penetrant fungicide with translaminar mobility, which means that it is absorbed by leaves and can move across the leaf to the opposite side of contact (Adama® 2021a). Although its action is mainly preventive, it also presents curative and anti-sporulation effects (Adama® 2021a). Tebuconazole is a triazol that inhibits the biosynthesis of sterol at demethylation (Lewis et al. 2016), thus interfering with the building of the fungal cell wall; it is a systemic fungicide with preventive, curative and eradicant effects (Kang et al. 2001; Lewis et al. 2016).

This study was structured in three stages tackling a total of five specific objectives. The first stage was focused on the assessment of the potential environmental impacts of azoxystrobin and tebuconazole. It specifically intended (i) to compare the sensitivity of non-target aquatic organisms to each fungicide by applying the Species Sensitivity Distributions (SSDs) approach; and (ii) to estimate the hazardous concentration for 5 and 50% of the species (HC_5_ and HC_50_) of aquatic ecosystems (micro- and macroinvertebrates, algae, plants, fish and amphibians) by the use of SSD models. Then, an organism sensitive to both fungicides was selected for representing the non-target species in the second stage of the work based on the information provided by the SSDs. This second stage specifically intended (iii) to infer about the safety of mixtures of these fungicides to non-target aquatic organisms. After fully characterizing the responses of the non-target species to the mixture of the AIs (modelled response surfaces validated experimentally), the third stage of the study was onset and aimed at (iv) evaluating the efficacy of mixtures of these fungicides at equivalent *vs*. alternative ratios and rates as in the commercial fungicide Custodia® against the target fungal species *Pyrenophora teres* CBS 123929 and *Rhynchosporium secalis* CBS 110524; and (v) appraising for a possible contribution of the other co-formulants used in Custodia® to the whole formulation toxicity. The overarching research hypothesis supporting the study is that the comercial pesticide products might be optimized during design stages to produce formulations effective against the target pests, but with the least possible impacts to the environment as indicated by sensitive non-target organisms. Changes in the ratio and/or combination of AIs and other co-formulants, as well as corresponding application rates represent prompt solutions to accomplish this purpose.

## Material and methods

### Chemicals

The commercial fungicide Custodia® (Adama®, Portugal) that combines the AIs azoxystrobin (120 g L^−1^; CAS: 131860-33-8) and tebuconazole (200 g L^−1^; CAS: 107534-96-3), as well as the analytical standards of these AIs (Merck®, Pestanal®, Steinheim), were comparatively tested in this study. The test solutions were prepared in deionized water, test medium or in acetone in case of insolubility in water at tested rates (efficacy assay with the target fungi), as specified below according to the requests of each experimental trial.

### Experimental approach

**Fig. 1:**
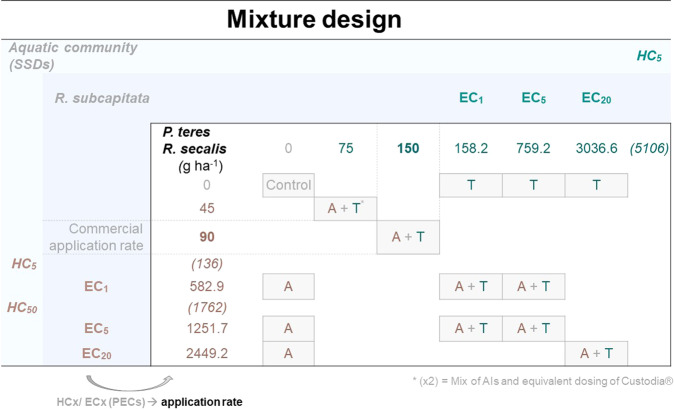
Mixture test design applied for assessing the efficacy of equivalent and alternative application rates of azoxystrobin (A) and tebuconazole (T) towards the target fungi *Pyrenophora teres* CBS 123929 and *Rynchosporium secalis* CBS 110524. ECx/HCx and a commercial application rate of these fungicides were included to relate effectiveness (target fungi) with environmental safety considering a non-target aquatic representative (*Raphidocelis subcapitata*) and aquatic communities as a whole (HCx estimated in the SSDs approach), respectively. Predicted environmental concentrations (PECs) equal to the estimated ECx were converted to application rates (AR; g ha^−1^)

A three-stages approach was followed in this study. The sensitivity of aquatic organisms (micro- and macroinvertebrates, algae, plants, fish and amphibians) to the fungicides azoxystrobin and tebuconazole was first evaluated. Namely, SSDs were built to appraise on the sensitivity of aquatic communities as a whole to each fungicide. Then, the corresponding standard benchmarks HC_5_ and HC_50_ were calculated (see section 2.2.1 for details). In addition, given that AIs might interact when mixed, and that a potentiation of the effects represents one of the possible outcomes, mixtures of azoxystrobin with tebuconazole were further tested in a selected non-target aquatic representative at the second stage of this study (see section 2.2.2 for details). *Raphidocelis subcapitata* (formerly known as *Pseudokirchneriella subcapitata*) was chosen considering its relative sensitivity to both herbicides (as pictured by the SSDs), responsivity in general to fungicides among standard aquatic organisms, and its recommendation for the assessment of the impact of pesticides to surface water organisms (EFSA 2013). Lastly, the efficacy of equivalent and alternative fungicide formulations towards two fungal species targeted by the selected commercial formulation was assessed (*P. teres* CBS 123929 and *R. secalis* CBS 110524; Fig. 1; see section 2.2.3 for details). Despite HC*x* are perhaps the most reliable benchmarks supporting environmental protection, the effect concentrations (EC*x*) estimated for *R. subcapitata* were alternatively considered when planning the mixture test design to assess safer alternative formulations. Specifically, the high application rates of each fungicide tested corresponded to levels of effect up to the EC_20_ in *R. subcapitata*. SSD information is not always available to support formulation design. Moreover, testing the effects of mixtures with as many organisms as those needed to compose a feasible SSD model is not cost-efficient, and hence not realistic in practice. Thus, the use of a representative non-target organism is a reasonable approach for establishing guiding environmentally protective benchmarks to frame the testing of alternative combinations of the fungicides as to their efficacy against the targets.

#### Assessing the sensitivity of non-target aquatic organisms to azoxystrobin and tebuconazole

SSDs reflecting short-term sublethal and lethal responses of aquatic organisms to azoxystrobin and tebuconazole were built based on median effect concentrations (EC_50_), either collected from the literature or calculated following ecotoxicological tests performed in the present study as needed to improve the overall model fitting of derived curves. Standard producers and primary consumers were selected for testing and further SSD inclusion given their important role in trophic chains, namely: *Lemna gibba*, *L. minor*, *Daphnia magna, Thamnocephalus platyurus* and *Brachionus calyciflorus*.

The aquatic plants *L. gibba* and *L. minor* were maintained in the laboratory in Steinberg medium (OECD 2006) at 20 ± 2 °C and 16 h^L^:8 h^D^ photoperiod. Growth inhibition tests with these macrophytes followed the OECD guideline 221 (OECD 2006) adapted to the use of 6-well plates (Kaza et al. 2007). Specifically, 3 healthy colonies with 3 fronds each were inoculated *per* well containing Steinberg medium (controls) or azoxystrobin/tebuconazole dissolved in Steinberg medium at defined concentrations (Table S1) in triplicate. The plates were incubated at 23 °C, for 7 days under continuous illumination. After the 7-days exposure period, the biomass yield was calculated as the difference between the inoculating and final frond number/dry weight.

Monoclonal bulk cultures of the crustacean *D. magna* were maintained in the laboratory in ASTM hard water (ASTM 1980) enriched with an organic extract of *Ascophyllum nodosum* at 20 ± 2 °C and a 16 h^L^:8 h^D^ photoperiod. Cultures were renewed 3 times *per* week and fed with the microalgae *R. subcapitata* (3.0 × 10^5^ cells mL^−1^). Juveniles at the 4^th^ instar (4–5 days old) were used in feeding inhibition tests as adapted from McWilliam & Baird (2002) and Allen et al. (1995). Specifically, 5 juveniles were added to each borosilicate flask containing ASTM plus algal food at a concentration of 3.0 × 10^5^ cells mL^−1^ (controls) or tebuconazole dissolved in ASTM plus the algal food (concentrations in Table S1), in quadruplicate. The flasks were incubated for 24 h at 20 ± 2 °C, in the dark. The feeding rates were calculated as the difference in microalgae density at 24 h compared to the beginning of the assay. Feeding inhibition tests were also run with *T. platyurus* by using the Rapidtoxkit F Thamno (MicroBioTests Inc.) according to the manufacturer’s instructions. Briefly, ≈36 h-larvae (fully developed digestive system) were added in duplicate to the control and test solutions at the defined test concentrations (Table S1); after 1-h incubation at 25 °C in the dark, a suspension of red microspheres was added for a 30-min feeding period, and particle uptake inhibition was calculated. The lethality of *T. platyurus* following a 24-h exposure period to azoxystrobin and tebuconazole was additionally assessed through the THAMNOTOXKIT F (MicroBioTests Inc.). Briefly, young larvae were incubated for 24 h at 25 °C, in the dark, to assess mortality following exposure to Standard Freshwater (controls) or the fungicide solutions prepared in Standard Freshwater (concentrations in Table S1). Finally, reproduction inhibition tests with the rotifer *B. calyciflorus* were performed through the ROTOXKIT F (MicroBioTests Inc.). Specifically, organisms hatched from cysts and fed with a Roti-rich food suspension were incubated individually (8 replicates *per* treatment) in Standard Freshwater plus algal food (controls) or tebuconazole solution prepared in Standard Freshwater plus algal food (concentrations in Table S1) for 48 h at 25 °C, in the dark; after a 48-h exposure period, dead and living rotifers were counted under the stereoscope (Olympus SZX9) for calculation of population growth rate inhibition.

#### Assessing the environmental safety of combinations of azoxystrobin and tebuconazole

The microalga *R. subcapitata* was selected as the representative non-target organism for mixture toxicity testing with azoxystrobin and tebuconazole. *R. subcapitata* was maintained in the laboratory in Woods Hole MBL medium (Nichols 1973), at 20 ± 2 °C and 16 h^L^:8h^D^ photoperiod. Growth inhibition tests were performed according to the OECD guideline 201 (OECD 2011) adapted to 24-well microplates (Geis et al. 2000). Specifically, *R. subcapitata* at a cell density of 10^4^ cell mL^−1^ was exposed in triplicate to single and mixture treatments of azoxystrobin and tebuconazole prepared in MBL (concentrations in Table S1) or to MBL only (controls) for 96 h at 23 ± 1 °C and continuous illumination. The concentrations tested in the mixture assay (Table S1) were defined following single-chemical exposures. One of the conditions tested consisted of the commercial formulation Custodia® diluted to concentrations of AIs equivalent to another mixture treatment comprising only the AIs at the same ratio. After the 96-h exposure period, the biomass yield (cells mL^−1^) was calculated as the difference between cell densities at the end and at the beginning of the assay.

#### Assessing the efficacy of combinations of azoxystrobin and tebuconazole against target fungal species

*Pyrenophora teres* CBS 123929 (isolated from leaf of barley in Hungary) and *Rhynchosporium secalis* CBS 110524 (isolated from barley cultivar Pipkin in England) were obtained from the CBS collection of the Westerdijk Fungal Biodiversity Institute (Netherlands). Both were cultured in the laboratory in Petri plates containing 3% (w/v) Malt Extract Agar (MEA) medium, at 21 °C ± 1 °C. The SSDs (section 2.2.1) and the results of the assay with the non-target representative (section 2.2.2) allowed to define the experimental mixture test design (Fig. 1) for the target fungi. Specifically, the EC_1_, EC_5_ and EC_20_ estimates for azoxystrobin and tebuconazole following testing with *R. subcapitata* were assumed to be environmentally safe levels defining the top range concentrations to be tested with the fungi; HC_5_ and HC_50_ derived from SSDs were also considered in the test design for comparison with the single organism approach. All these reference values are concentrations in water. For a realistic definition of its correspondence with pesticide application rates and consequent testing with fungi, we assumed ECx/HCx as Predicted Environmental Concentrations (PECs) of the fungicides in surface water, and converted them to application rates by using standard European tools for pesticide risk assessment (FOCUSsw European tool; Linders et al. 2001). Only lower steps (Steps 1/2) of this tool were applied since specific transport models/scenarios were not herein considered. Simulations regarding different application rates allowed to define regressions between application rates and PECs at 0 days post-application (maximum concentrations; parameters used in the simulations presented in Table S2) for azoxystrobin (Eq. 1; *n* = 9, r^2^ = 1.00) and tebuconazole (Eq. 2; *n* = 9 with r^2^ = 1.00), as follows:

1 $${{{\mathrm{PEC}}}}_{{{{\mathrm{surface}}}}\,{{{\mathrm{water}}}}}\left( {{{{\mathrm{EC}}}}_{\it{X}}{{{\mathrm{or}}}}\,{{{\mathrm{HC}}}}{\it{x}}} \right) = 0.2213 \times {{{\mathrm{application}}}}\,{{{\mathrm{rate}}}}\,{{{\mathrm{in}}}}\,{{{\mathrm{target}}}}\,{{{\mathrm{fungi}}}}$$

2 $${{{\mathrm{PEC}}}}_{{{{\mathrm{surface}}}}\,{{{\mathrm{water}}}}}\left( {{{{\mathrm{EC}}}}_X{{{\mathrm{or}}}}\,{{{\mathrm{HC}}}}x} \right) = 0.1736 \times {{{\mathrm{application}}}}\,{{{\mathrm{rate}}}}\,{{{\mathrm{in}}}}\,{{{\mathrm{target}}}}\,{{{\mathrm{fungi}}}}$$

Growth inhibition tests with the two fungal species were performed as in Gonçalves et al. (2020). Briefly, 5-mm-diameter plugs of the mycelium of actively growing colonies were placed in triplicate in 90-mm assay plates containing 3% (w/v) MEA medium (controls) or MEA medium supplemented with azoxystrobin and tebuconazole (concentrations in Table S3). Specifically, stock solutions of azoxystrobin and tebuconazole prepared in acetone (1% max. final concentration) were spiked singly or in mixture into a constant volume of sterile deionized water (*i.e*. 0.38 mL that corresponds to the volume of pesticide preparation as recommended in Custodia® *–* 600 L ha^−1^; note that the area of the test plate is equivalent to 6.3585e^−07^ ha), which was spread onto the surface of the solidified agar medium for a final total volume equal to 20 mL. A control for acetone at the maximum concentration applied was run in parallel. The plates were incubated at 21 °C ± 1 °C for 21 days, in the dark. At the end of the exposure period, the mean diameter *per* condition was recorded and the growth inhibition comparatively to controls was calculated.

### Data analysis

The data obtained in the single assays with non-target organisms were used to estimate corresponding EC_50_ values by using regression models (non-linear regression for biomass yield and population growth rate; probit regression for feeding inhibition and lethality). The EC_50_ values for azoxystrobin and tebuconazole estimated herein or collected from the literature (Table S4) were then used to build SSDs by using the SSDs generator from EPA (US EPA 2021). The SSD models allowed to estimate the hazardous concentrations affecting 5 and 50% of the species (HC_5_ and HC_50_, respectively). Regarding mixture toxicity analysis, the data obtained in the assay with *R. subcapitata* were compared to the reference Concentration Addition and Independent Action mixture models, and deviation functions *– i.e*. synergism/antagonism (S/A), dose-level (DL) or dose-ratio (DR) dependent effects *–* to identify the model that best described the mixture behavior (*e.g*. Jonker et al. 2005). This analysis was run in a customized MS®Excel® spreadsheet (ToxCalcMix, version 1.0, last rev. 20/01/2016; AJA Nogueira, unpublished), as explained in detail by Queirós et al. (2018). The data obtained in the mixture assays with both the non-target (*R. subcapitata*) and target (*P. teres* CBS 123929 and *R. secalis* CBS 110524) representatives were additionally analyzed via one-way ANOVA, followed by the post-hoc Tukey’s test, to assess significant differences among tested conditions after assumptions verification (Anderson-Darling test and Levene’s test). A Student’s t-test was also run for the comparison of mean diameters of the target fungi in the regular (water) and solvent (acetone) controls. A significance level of 0.05 was always used.

## Results and discussion

### Species sensitivity distributions for azoxystrobin and tebuconazole

**Fig. 2:**
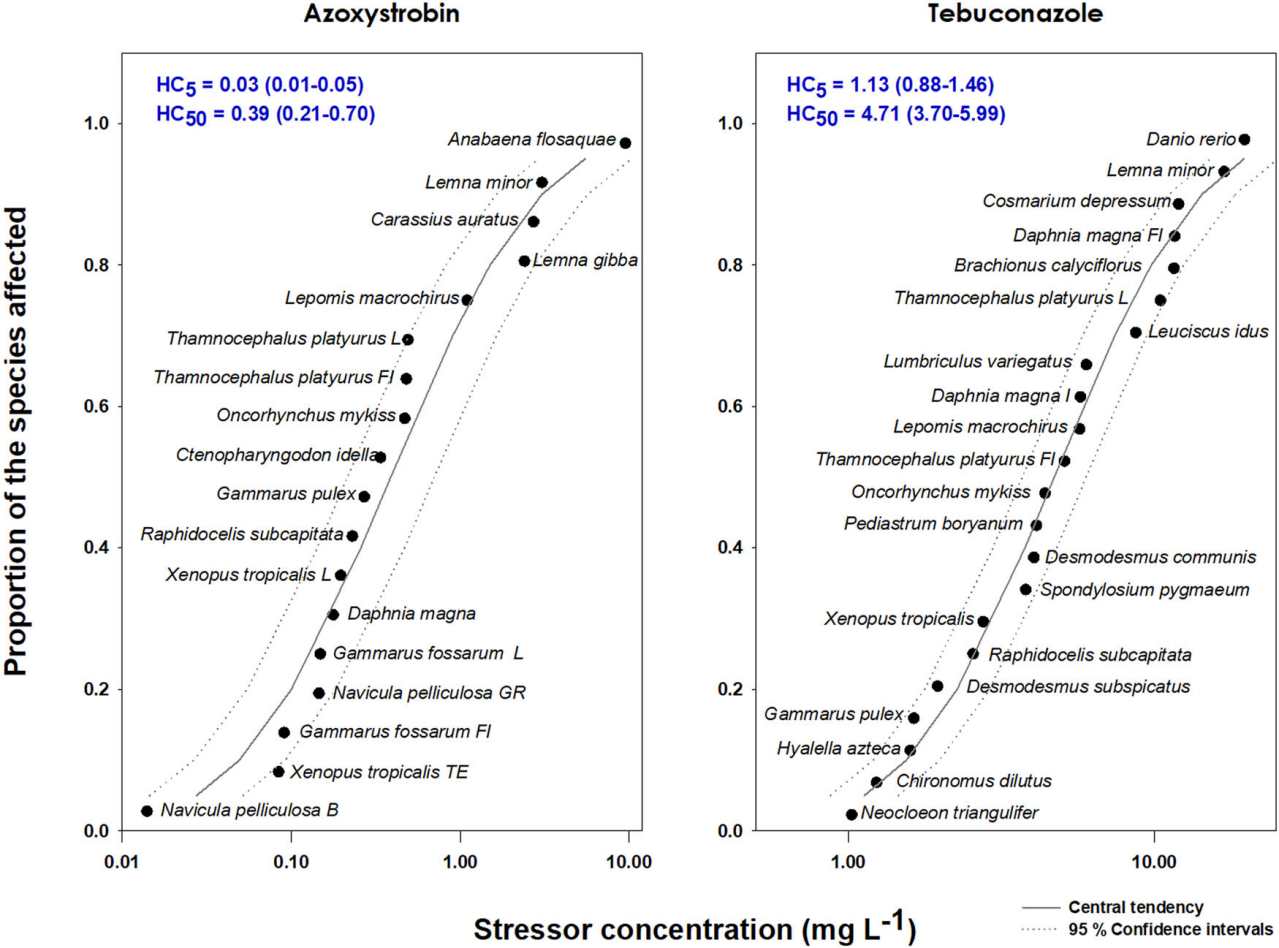
Species Sensitivity Distribution curves (SSDs) for azoxystrobin and tebuconazole built with EC_50_ values (Table S4) estimated from short-term toxicity assays with aquatic organisms. The central lines represent the SSD model fitted to the data regarding each fungicide (R^2^ = 0.960 for azoxystrobin; R^2^ = 0.976 for tebuconazole) and the dotted lines represent corresponding 95% confidence intervals

The generated SSDs and corresponding HC*x* estimates (Fig. 2) indicate that aquatic organisms are more sensitive to azoxystrobin than to tebuconazole with little uncertainty given the very good model fitting found for both SSDs (R^2^ = 0.960 and 0.976 for azoxystrobin and tebuconazole, respectively). Specifically, the HC_5_ values calculated for azoxystrobin and tebuconazole (HC_5_ = 0.03 and 1.13 mg L^−1^, respectively) present a difference of two orders of magnitude. There was not a consistent pattern in the sensitivity distribution within each SSD generated in the present work. For example, different species of crustaceans and algae are found both among the organisms with high (the crustaceans *Gammarus fossarum* and *D. magna*, and the diatom *Navicula pelliculosa*) and intermediate sensitivity (the crustaceans *Gammarus pulex* and *T. platyurus*, and the microalgae *R. subcapitata*) to azoxystrobin. Regarding tebuconazole, different algae species are found among the organisms with high (*Desmodesmus subspicatus* and *R. subcapitata*), intermediate (*Spondylosium pygmaeum*, *D. communis* and *Pediastrum boryanum*) and low (*Cosmarium depressum)* sensitivity to this fungicide. The diatom *N. pelliculosa* and the mayfly *Neocloeon triangulifer* were the most sensitive to azoxystrobin and tebuconazole, respectively. Macrophytes (genus *Lemna*) were among the organisms with lower sensitivity to both fungicides. Although *R. subcapitata* (the non-target representative selected for mixture toxicity testing to infer about the environmental safety of azoxystrobin and tebuconazole combinations) was not the most sensitive to both herbicides, it is at the first half of both SSD curves, similarly to other algal species. In addition to *R. subcapitata*, the crustacean *Daphnia magna* and the fish *Oncorhynchus mykiss* are also model species highly recommended in the assessment of pesticide impacts (including specifically the fungicides group) to surface water ecosystems (EFSA 2013). While *D. magna* seemed to be more sensitive to azoxystrobin than the green microalga, the opposite occurred regarding tebuconazole. The fish *Oncorhynchus mykiss* seemed to be less sensitive than *R. subcapitata* regarding both fungicides.

The absence of a consistent pattern in the sensitivity degree observed for the different groups of organisms included in the SSDs is possibly linked to the general nature of the mechanisms of toxic action of these fungicides. Both are related to the inhibition of processes that occur in all eukaryotes (*i.e*. azoxystrobin – mitochondrial respiration; tebuconazole – sterol biosynthesis). For instance, previous studies showed that azoxystrobin caused mitochondrial dysfunction in distinct organisms like the zebrafish *Danio rerio* (Cao et al., 2018), the microalgae *Chlorella vulgaris* and the midge *Chironomus dilutes* (Wei et al., 2021), along with other observed effects like oxidative stress. Accordingly, tebuconazole provoked alterations in the levels of cholesterol in the zebrafish (Sancho et al., 2010), and disrupted steroidogenesis in the frog *Xenopus laevis*.

While detected levels of tebuconazole in environmental aquatic systems seem to be below the calculated HC_5_ (maximum of 9.1 µg/L; Battaglin et al. 2011; Berenzen et al. 2005; Kahle et al. 2008), maximum concentrations of azoxystrobin equal to the respective HC_5_ were detected in runoff-triggered water samples from small streams in Germany (29.7 µg/L; Berenzen et al. 2005; Rodrigues et al. 2013). This suggests that a potential risk for aquatic communities should be considered, especially relevant regarding benthic diatoms (*e.g. N. pelliculosa*; Fig. 2). Importantly, diatoms have an enormous ecological relevance as primary producers and also as biostabilizers of the sediments in riverine ecosystems (Santos et al. 2021). Thus, the authorization or conditions of use of azoxystrobin should probably be revised. Additionally, the risk of these fungicides to non-target aquatic fungi, which have important ecological roles in aquatic environments such as degradation of organic dead material (Ittner et al. 2018), should be evaluated in further studies. For instance, EC_100_ values lower than 10 mg L^−1^ were estimated for the non-target fungi *Fusarium sporotrichioides* and *Trichoderma hamatum* (4.1 and 8.2 mg L^−1^ tebuconazole, respectively), and *Pythium spp*. isolates (0.1 and 5.0 mg L^−1^ azoxystrobin for the two tested isolates), in a study from Dijksterhuis et al. (2011), which focused on the effects of several fungicides on fungal growth.

### Mixtures of azoxystrobin and tebuconazole are safer to *R. subcapitata* than corresponding single treatments

**Fig. 3:**
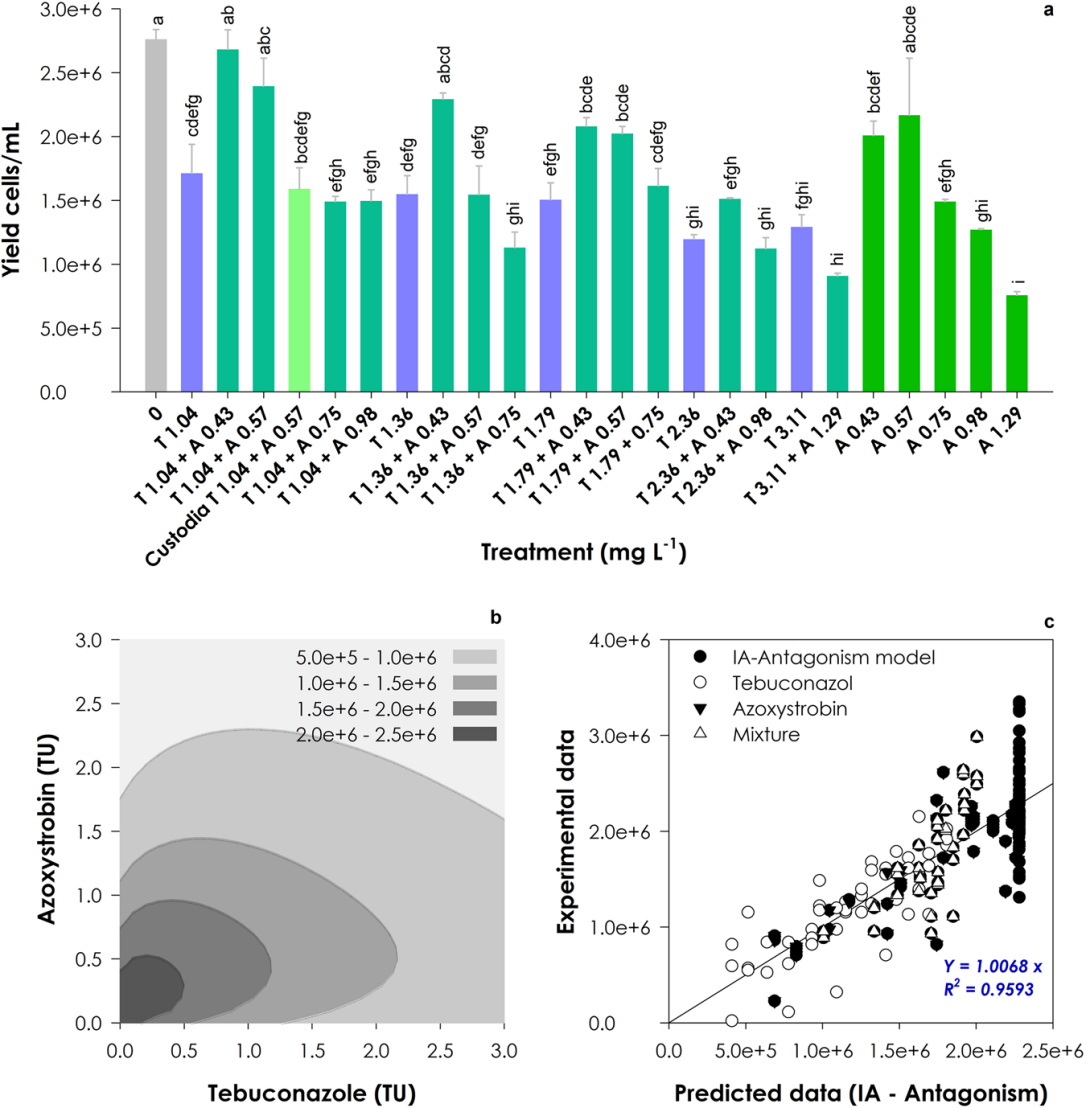
Response of *Raphidocelis subcapitata* measured following 96 h of exposure to single and mixture treatments of tebuconazole (T) and azoxystrobin (A). The bars graph (**a**) shows the average yield (*n* = 3; error bars represent the standard error) and significant differences among treatments are assigned using low case letters (Tukey test, *p* < 0.05). The isobologram (**b**) illustrates the mixture response surface as predicted by the IA model with an antagonistic type of deviation, using a TU dimensionless strength scaling (TU is the concentration in the mixture normalized to the single-chemical EC_50_) and a grey-scale gradient indicative of the level of the effect (the lighter the colors, the lower the yield). The scatter plot (**c**) shows the regression between experimental data and data predicted by the IA – Antagonism model, disclosing the respective equation and coefficient

All single treatments of tebuconazole tested in the mixture assay significantly inhibited the growth of *R. subcapitata* compared to the control group (Fig. 3a; F_24, 66_ = 25.61, *p* < 0.001). However, no significant differences were found among single tebuconazole treatments, with the calculated percent inhibition in yield varying shortly between 38% and 53%. Considering azoxystrobin, only the second lowest concentration tested resulted in a non-significant decrease in growth. In the other treatments, the growth inhibition was directly proportional to the concentration of azoxystrobin tested. In particular, the growth of *R. subcapitata* was negatively affected the most by the highest concentration of azoxystrobin (1.29 mg L^−1^) and the mixture including the highest concentration of both fungicides (3.11 mg tebuconazole L^−1^ added to 1.29 mg azoxystrobin L^−1^). The treatment where the commercial formulation Custodia® was dosed (1.04 mg tebuconazole L^−1^ added to 0.57 mg azoxystrobin L^−1^; Fig. 3a, light green bar), apparently affected more the growth of the microalga than the equivalent treatment including the mixture of the AIs at the same concentrations. Although this difference was not statistically significant, it suggests that a contribution of the other formulants to the overall commercial formulation’s toxicity cannot be ruled out. In fact, information was found in the literature suggesting high toxicity of 1,2-benzisothiazol-3(2H)-one (one of the specified co-formulants of Custodia®) to microalgae (Wang et al., 2018).

In general, the mixtures of azoxystrobin and tebuconazole were less toxic to *R. subcapitata* than the corresponding single treatments of the fungicides, as depicted in Fig. 3a and corroborated by the modelling of mixture toxicity data. Antagonism occurred throughout the whole mixture response surface, as clearly illustrated by the convex curves of the corresponding isobologram (Fig. 3b) built on the basis of the model that best fitted to the data (IA with antagonism denoted by *a* > 0; see Table 1) ‒ the association between the experimental data and predictions by the IA-antagonism model was indeed very strong (R^2^ = 0.9593, Fig. 3c). The better adjustment of the IA reference model to the mixture data was theoretically expected, given the distinct modes of toxic action of the two fungicides. However, the antagonistic interaction between the two fungicides, which denotes ‘a protective effect’ of this mixture towards the non-target microalgae compared to single exposures, is somewhat surprising. In principle, the AIs are combined in commercial pesticides to improve the efficacy against the targets. It is true that this result regards non-target microalgae, but both algae and fungi share the involved metabolic pathways (*i.e*. mitochondrial respiration and sterol biosynthesis; Lewis et al. 2016; NCB National Center for Biotechnology (2021)). Further studies would be important to clarify the specific mechanisms involved in this antagonistic interaction.

**Table 1:** Statistical parameters resulting from the fitting of experimental data to the reference models of mixture toxicity for Concentration Addition (CA) and Independent Action (IA), as well as to deviations from these models, i.e. dose-level dependence (DL), synergism/antagonism (S/A) and dose-ratio (DR)

		*r*^2^	RMSD	SSE (df)	AIC	*P* (F-test)	a	bDL	B_azox_	b_teb_
CA	Baseline	—	—	3.070E + 13 (33)	1082.80	—	—	—	—	**—**
	DL	0.526	384445.226	5.470E + 12 (31)	1021.49	<0.001	10.78	0.29	—	**—**
	S/A	0.435	414122.165	6.520E + 12 (32)	1025.23	<0.01	6.11	—	—	**—**
	DR	0.463	414837.924	6.200E + 12 (30)	1029.67	<0.01	4.08	—	6.52	−2.43
**IA**	Baseline	**—**	**—**	2.48E + 13 (33)	1074.42	**—**	**—**	**—**	**—**	**—**
	DL	0.568	366785.923	4.98E + 12 (31)	1017.83	<0.001	7.05	0.87	**—**	**—**
	**S/A**	**0.505**	**387770.503**	**5.71E** + **12 (32)**	**1020.10**	**<0.001**	**3.44**	**—**	**—**	**—**
	DR	0.524	390533.179	5.49E + 12 (30)	1024.96	<0.01	2.31	**—**	3.46	−1.16

Data regards the mixture of azoxystrobin (azox) and tebuconazole (teb) tested with *Raphidocelis subcapitata*. RMSD (Root Mean-Square Deviation) provides a measure of the difference between predicted and obtained values; SSE (Sum of Squared Errors) is the sum of the squared differences between each observation and its group’s mean, and df refers to the residual degrees of freedom; AIC (Akaike’s Information Criterion) expresses model fit (the lower the better); a, b_DL_, b_azox_ and b_teb_ are parameters of the deviation functions (functions presented in Jonker et al. 2005). Bold is used to highlight the model that best fitted the dataset regarding all the assessed parameters

### The combination of azoxystrobin and tebuconazole does not seem to represent an effective option to prevent the growth of the target fungi

**Fig. 4:**
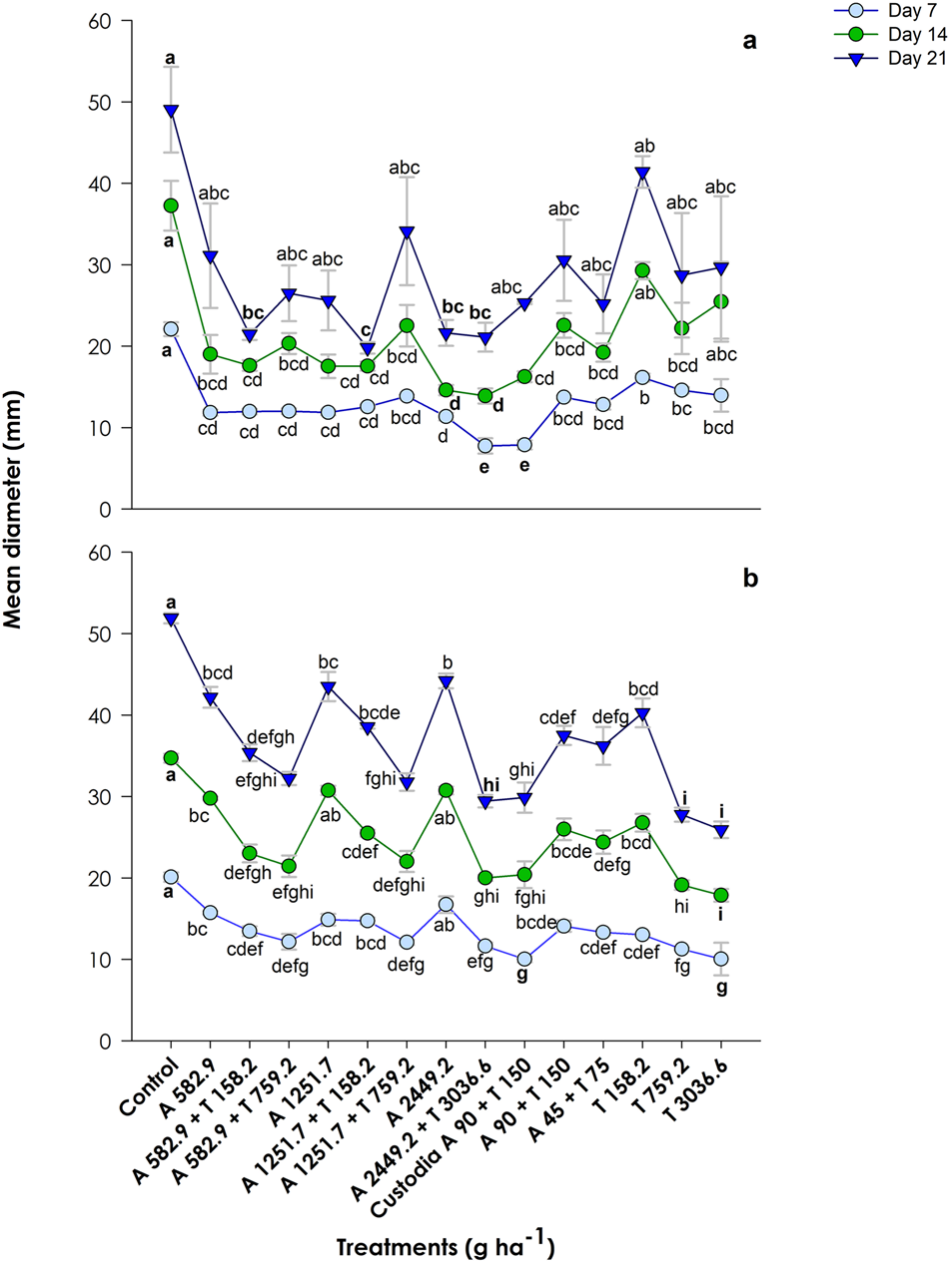
Growth response of the target fungi *Pyrenophora teres* CBS 123929 (**a**) and *Rynchosporium secalis* CBS 110524 (**b**) to single and mixture treatments of azoxystrobin (A) and tebuconazole (T), or to Custodia® diluted to the same concentration of active ingredients tested in one of the mixture treatments. The growth response is presented in terms of average mean diameter ± standard error (*n* = 3) of the fungi mycelium at 7, 14 and 21 days of exposure. Low case letters are used to assign significant differences between conditions within each assessed timepoint (post hoc Tukey test, *p* < 0.05). Lines were added only for visualization purposes

The *t*-tests showed that the mean diameters of the target fungi in the acetone controls were not significantly different from those in the water controls (*p* > 0.05) regarding both fungal species and the three assessed timepoints (days 7, 14 and 21). Consistently with the use of solvents in the treatments, the solvent control was used for further data visualization and statistical analysis (one-way ANOVA). Regardless of application rates, single and combined dosing of azoxystrobin and tebuconazole, as well as the commercial formulation Custodia®, significantly affected the growth of *P. teres* CBS 123929 at day 7 compared to the control group (Fig. 4a; F_14, 31_ = 29.59, *p* < 0.001). However, the mean diameter of the fungal mycelium at day 14 was no longer significantly different from the control in two of the tested conditions (*i.e*. 158.2 and 3036.6 g ha^−1^ of tebuconazole; F_14, 31_ = 7.83, *p* < 0.001). At day 21, this was the case for most of the tested conditions (differences compared to the control were still found except for 582.9 g ha^−1^ of azoxystrobin added to 158.2 g ha^−1^ of tebuconazole; 1251.7 g ha^−1^ of azoxystrobin added to 158.2 g ha^−1^ of tebuconazole; 2449.2 g ha^−1^ of azoxystrobin and 2449 g ha^−1^ of azoxystrobin mixed with 3036.6 g ha^−1^ of tebuconazole; F_14, 30_ = 3.22, *p* < 0.01). Overall, the conditions negatively affecting the most *P. teres* CBS 123929 at day 21 caused limited maximum mean growth inhibition, ranging within 62–71%. Furthermore, mixture treatments were generally not differentially effective than counterpart single fungicide treatments in controlling *P. teres* CBS 123929. A noticeable exception was found for the mixture including the maximum concentration of each AI (2449 g azoxystrobin ha^−1^ mixed with 3036.6 g tebuconazole ha^−1^), which was slightly more effective than the corresponding single treatments yet only at the 7th day of exposure. This combined treatment was one of the most effective options to control *P. teres* CBS 123929 at day 7 (growth inhibition >65%), along with the commercial formulation at much lower application rates. However, equivalent or even higher mean growth inhibition rates were later recorded at days 14 and 21 under other experimental treatments (*e.g*. 2449.2 g azoxystrobin ha^−1^ at days 14 and 21, and 1251.7 g azoxystrobin ha^−1^ combined with 158.2 g tebuconazole ha^−1^ at day 21).

Considering *R. secalis* CBS 110524 (Fig. 4b), all tested fungicide conditions, except for the highest concentration of azoxystrobin (2449.2 g ha^−1^), significantly inhibited fungal growth at day 7 compared to the control group (F_14, 29_ = 21.97, *p* < 0.001). At day 14, the response pattern was very similar (F_14, 29_ = 27.56, *p* < 0.001), but an additional exception corresponding to azoxystrobin dosed at 1251.7 g ha^−1^ was noted. At day 21, all tested conditions reduced significantly *R. secalis* CBS 110524 growth compared to the control group (F_14, 26_ = 29.87, *p* < 0.001). Even though, the highlighted conditions corresponding to single azoxystrobin treatments, as well as the treatments dosing both the AIs singly at the lowest concentrations, were the least effective in inhibiting the growth of *R. secalis* CBS 110524 (up to a maximum of 13–21% mean growth inhibition). No mixtures were significantly more effective than the corresponding single AI dosing in controlling *R. secalis* CBS 110524. Generally, exposure to single rates of tebuconazole resulted in similar or even lower mean mycelium diameter than observed in the corresponding mixtures. Overall, the conditions that caused higher growth inhibitions in *R. secalis* CBS 110524 at day 21 were the two highest single rates of tebuconazole (759.2 and 3036.6 g ha^−1^), yet reflecting in limited growth inhibitions of 45 and 49%, respectively. The commercial formulation was indeed one of the most effective conditions regarding fungal growth inhibition at day 7 (growth inhibition of 49%), but other treatments caused equivalent or higher mean growth inhibitions than that of Custodia® as assessed later at days 14 and 21.

In general, both non-formulated and formulated (Custodia®) forms of the fungicides azoxystrobin and tebuconazole were more effective in preventing the growth of *P. teres* CBS 123929 (mean growth inhibitions of 29–66% for day 7, 39–71% for day 14, and 39–71% for day 21) than *R. secalis* CBS 110524 (mean growth inhibitions of 15–49% for day 7, 14–50% for day 14, and 13–49% for day 21). Specifically, the mean growth inhibition caused by the commercial product was around 60–70% in *P. teres* CBS 123929, but 40–50% in *R. secalis* CBS 110524 for all the assessed timepoints. While higher mean growth inhibitions were found for days 14 and 21 compared to day 7 regarding *P. teres* CBS 123929 (*i.e*. the action of the fungicides increased at the second week and kept constant over the third week, except for the commercial product and mixture including the highest concentration of both fungicides), those values did not vary over time for *R. secalis* CBS 110524. These differences in sensitivity can be related to the interaction of the toxic with the distinct species, for instance differences in internalization, transport or bioavailability. The other formulants of Custodia® seem hence to have an effective action (*i.e*. potentiation of the effects of the AIs by ≈ 25%) in the overall toxicity of the AIs regarding also the target fungi *–* treatment with Custodia® *vs*. treatment with the corresponding mixture of the AIs at the same rates (Fig. 4). Specifically, the statistical analysis pointed out significant differences between these treatments in at least one (*P. teres* CBS 123929 *–* day 7) or all the assessed timepoints (*R. secalis* CBS 110524). This suggests either that: (i) the other formulants of Custodia® enhanced the action of the AIs in the tested organisms, for instance by improving its uptake/translocation (*e.g*. Castro et al. 2014); (ii) these supposedly inert formulants were toxic at applied concentrations (*e.g*. Adams et al. 2021; Mesnage et al. 2013); (iii) or even both (*e.g*. Karaca et al. 2021). For instance, propane-1,2-diol is frequently used as solvent (*e.g*. NCB National Center for Biotechnology (2022a)) and might have contributed to improve the uptake of the fungicides. In addition, 1,2-Benzisothiazol-3(2H)-one presents antifungal activity (Borgna et al. 1996), and glyoxal also has a role as pesticide (*e.g*. NCB National Center for Biotechnology (2022b)).

While *R. subcapitata* and the other non-target aquatic organisms were in general more sensitive to azoxystrobin than to tebuconazole (*e.g*. Fig. 2), this pattern was not evident regarding the target fungi. In fact, data suggest the opposite respecting *R. secalis* CBS 110524 (*e.g*. A 2449.6 g ha^−1^ *vs*. T 759.2 g ha^−1^ in Fig. 4). Despite an increase of more than 4-fold in the application rates of azoxystrobin and tebuconazole in the target fungus *P. teres* CBS 123929, significant differences were not found between treatments including at the highest concentration of each fungicide. The same occurred also for azoxystrobin regarding the other fungal species. In theory, low concentrations of fungicide can trigger stress response mechanisms allowing the fungi to survive, but then high concentrations would overwhelm these responses causing death. Nevertheless, high concentrations might have activated different mechanisms causing tolerance (Hayes et al. 2014). The mixture of AIs with different modes of action is a strategy used to broaden the spectrum of action of commercial pesticides (*i.e*. for controlling more pests), to improve the disease control, and to circumvent resistance (van den Bosch et al. 2014). Remarkably, herein the mixture of azoxystrobin with tebuconazole has not shown to be more effective than the corresponding single treatments in the growth control of the two target fungal species. Literature reports that this mixture of AIs in a commercial product was effective against different fungi as *Pyricularia oryzae* (Mohiddin et al., 2021), *Golovinomyces cichoracearum* and *Podosphaera fusca* (Nosehy et al. 2019). Curiously, Nosehy et al. (2019) obtained very similar levels of efficacy against powdery mildew for a commercial formulation containing only azoxystrobin *vs*. a commercial product containing a mixture of azoxystrobin with tebuconazole at very similar rates of azoxystrobin applied (curative application: 61.2% *vs*. 57.6% efficacy; protective application: 76.6% *vs*. 70.7% efficacy, respectively for the azoxystrobin and mixture of azoxystrobin with tebuconazole); for a commercial formulation containing only tebuconazole at high rates of AI, the levels of efficacy were of only 42.7 and 55.9% for curative and protective application, respectively. The application rates recommended in Custodia® for the treatment of winter cereal diseases caused by fungi like *P. teres* CBS 123929 and *R. secalis* CBS 110524 (1–1.25 L ha^−1^ that corresponds to 120–150 g azoxystrobin ha^−1^ mixed with 200–250 g tebuconazole ha^−1^) translate into predicted concentrations of the AIs in the surface water that are apparently safe to the non-target species *R. subcapitata* (*i.e*. lower than the EC_1_ for azoxystrobin and between the EC_1_ and EC_5_ for tebuconazole), and to aquatic communities in general (lower than the HC_5_ for both fungicides; Fig. 1; Table S3). Nevertheless, the present study suggests that these recommended application rates of Custodia® might not be totally effective in the control of *P. teres* CBS 123929 and *R. secalis* CBS 110524. While close application rates tested herein towards the isolated fungi caused maximum growth inhibitions of 60–70% and 40–50% in *P. teres* CBS 123929 and *R. secalis* CBS 110524, both fungi continued growing overtime when treated with higher application rates of these AIs (Fig. 1). Despite the magnitude of growth had significantly changed among tested conditions (*e.g*. A1251.7 + T158.2 *vs*. T158.2 for *P. teres* CBS 123929, and T3036.6 *vs*. A2449.2 for *R. secalis* CBS 110524; Fig. 4), none of the tested fungicide treatments, including the commercial formulation at recommended application rates, satisfactorily prevented the proliferation of the target fungi. Importantly, an ineffective control of the fungi and continuous exposure to suboptimal fungicide concentration can potentially contribute to the development of resistance mechanisms (*e.g*. Perlin et al. 2017).

## Conclusions

None of the tested fungicide treatments, *i.e*. recommended and alternative application rates corresponding to environmentally safe levels of effect in non-target aquatic organisms, totally prevented the growth of fungi targeted by the commercial formulation. The maximum levels of inhibition achieved were around 70% in *P. teres* CBS 123929 and 50% in *R. secalis* CBS 110524 throughout the 21 days, which likely might favor the development of resistance mechanisms. The mixture of azoxystrobin with tebuconazole as used is Custodia® was not more toxic to the representative non-target organism (*R. subcapitata*) than the equivalent exposure to the single fungicides (antagonistic action – protective effect). Analogously, mixtures of these AIs were not more effective in the control of *P. teres* CBS 123929 and *R. secalis* CBS 110524 than the respective single treatments. Both the assays with the non-target microalgae and the target fungi suggested that the other formulants of Custodia® seem to have an effective action (*i.e*. potentiation of the effects of the AIs) in the overall product toxicity, possibly by provoking an increased AIs uptake, or by presenting inherent toxicity. Regarding the target fungi, the potentiation of the effects of the AIs by the co-formulants was more prominent at day 7, with an increase in the mean growth inhibition of about 25%. Overall, our results suggest that this type of formulation combining azoxystrobin with tebuconazole does not represent an effective option for treating these two fungal species although it seems to be environmentally safe.

## Supplementary information

SUPPLEMENTARY INFORMATION
