# Supplementary material for: Recommended rates of azoxystrobin and tebuconazole seem to be environmentally safe but ineffective against target fungi
Source: Ecotoxicology. 2023 Jan 18;32(1):102–13. doi: 10.1007/s10646-023-02619-w (PMC9883303; doi:10.1007/s10646-023-02619-w)
Supplement: Supplementary file 1 — SUPPLEMENTARY INFORMATION [file 10646_2023_2619_MOESM1_ESM.docx]

**SUPPLEMENTARY INFORMATION**

**A comparative safety-efficacy assessment of the fungicides azoxystrobin and tebuconazole considering the aquatic compartment and two target fungal species**

Libânia Queirós^(libania.queiros@ua.pt)^, Nuno Aguiar, Patrícia Pereira, Fernando J.M. Gonçalves, Artur Alves, Joana Luísa Pereira

**Table S1** – Concentrations/application rates of azoxystrobin (A) and tebuconazole (T) tested in the assays with the non-target organisms (*Lemna gibba*, *Lemna minor*, *Daphnia magna*, *Thamnocephalus platyurus*, *Brachionus calyciflorus* and *Raphidocelis subcapitata*; in mg L^-1^). The treatment corresponding to the commercial formulation Custodia® diluted to the same concentration of active ingredients as tested in another mixture treatment is presented in bold.

| **Non-target organisms’ exposures** | | | | | | | | |  | **Non-target representative’ exposure** | | | |  |
| --- | --- | --- | --- | --- | --- | --- | --- | --- | --- | --- | --- | --- | --- | --- |
| ***Lemna gibba*** | ***Lemna minor*** | | ***Daphnia magna*** | ***Thamnocephalus platyurus*** | | | | ***Brachionus calyciflorus*** |  | | ***Raphidocelis subcapitata*** | | |  |
| **A** | **A** | **T** | **T** | **A**  **(feeding)** | **T**  **(feeding)** | **A**  **(lethality)** | **T**  **(lethality)** | **T** |  | **A** | | **T** | **A + T** |  |
| mg L^-1^ | | | | | | | | |  | mg L^-1^ | | | |  |
| 0.00 | 0.00 | 0.00 | 0.00 | 0.00 | 0.00 | 0.00 | 0 | 0.00 |  | 0.00 | | 0.00 | 0.00 + 0.00 |  |
| 0.03 | 0.08 | 0.08 | 2.50 | 0.04 | 1.00 | 0.35 | 3.33 | 2.50 |  | 0.04 | | 0.70 | 0.00 + 1.04 |  |
| 0.04 | 0.11 | 0.13 | 3.38 | 0.06 | 1.40 | 0.39 | 5.16 | 5.68 |  | 0.05 | | 0.91 | 0.00 + 1.36 |  |
| 0.07 | 0.16 | 0.20 | 4.56 | 0.09 | 1.96 | 0.44 | 8 | 7.48 |  | 0.07 | | 1.18 | 0.00 + 1.79 |  |
| 0.11 | 0.23 | 0.33 | 6.15 | 0.14 | 2.74 | 0.49 | 12.41 | 9.83 |  | 0.10 | | 1.54 | 0.00 + 2.36 |  |
| 0.18 | 0.34 | 0.52 | 8.30 | 0.20 | 3.84 | 0.55 | 19.23 | 12.93 |  | 0.13 | | 2.00 | 0.00 + 3.11 |  |
| 0.28 | 0.49 | 0.84 | 11.21 | 0.30 | 5.38 | 0.62 |  | 17.00 |  | 0.18 | | 2.60 | 0.431 + 0.00 |  |
| 0.45 | 0.72 | 1.34 | 15.13 | 0.46 | 7.53 | 0.69 |  | 22.35 |  | 0.24 | | 3.38 | 0.568 + 0.00 |  |
| 0.72 | 1.04 | 2.15 | 20.43 | 0.68 | 10.54 |  |  | 29.39 |  | 0.33 | | 4.39 | 0.75 + 0.00 |  |
| 1.16 | 1.50 | 3.44 | 27.58 | 1.03 | 14.76 |  |  |  |  | 0.44 | | 5.71 | 0.98 + 0.00 |  |
| 1.86 | 2.18 | 5.50 |  |  |  |  |  |  |  | 0.60 | | 7.42 | 1.29 + 0.00 |  |
| 2.97 | 3.16 | 8.80 |  |  |  |  |  |  |  | 0.80 | | 9.65 | 0.431 + 1.04 |  |
| 4.75 | 4.59 | 14.07 |  |  |  |  |  |  |  | 1.09 | |  | 0.57 + 1.04 |  |
|  |  |  |  |  |  |  |  |  |  | 1.47 | |  | **0.57 + 1.04** |  |
|  |  |  |  |  |  |  |  |  |  |  | |  | 0.75 + 1.04 |  |
|  |  |  |  |  |  |  |  |  |  |  | |  | 0.98 + 1.04 |  |
|  |  |  |  |  |  |  |  |  |  |  | |  | 0.43 + 1.36 |  |
|  |  |  |  |  |  |  |  |  |  |  | |  | 0.57 + 1.36 |  |
|  |  |  |  |  |  |  |  |  |  |  | |  | 0.75 + 1.36 |  |
|  |  |  |  |  |  |  |  |  |  |  | |  | 0.43 + 1.79 |  |
|  |  |  |  |  |  |  |  |  |  |  | |  | 0.57 + 1.79 |  |
|  |  |  |  |  |  |  |  |  |  |  | |  | 0.75 + 1.79 |  |
|  |  |  |  |  |  |  |  |  |  |  | |  | 0.43 + 2.36 |  |
|  |  |  |  |  |  |  |  |  |  |  | |  | 0.98 + 2.36 |  |
|  |  |  |  |  |  |  |  |  |  |  | |  | 1.29 + 3.11 |  |

**Table S2.** Values used for the required parameters in Step 1 of the FOCUS Surface water EU tool* for estimating predicted environmental concentrations (PECs) in surface water, according with the application doses of each active substance. Substance specific chemical data was retrieved from EFSA reports for azoxystrobin (EFSA, 2010) and tebuconazole (EFSA, 2014).

|  | Azoxystrobin | Tebuconazole |
| --- | --- | --- |
| Water solubility (mg L^-1^) | 6.0 | 36.0 |
| K_OC_ /K_foc_ (L kg^-1^) | 427.0 | 769.0 |
| DT_50_ in soil (days) | 78.0 | 47.7 |
| DT_50_ in water/sediment system (days) | 205.0 | 365.0 |
| DT_50_ in water (days) | 1000.0 | 999.0 |
| DT_50_ in sediment (days) | 205.0 | 999.0 |
| Application pattern (crop, region and season of application, number of applications *per* season) | Cereals, Southern Europe, autumn-winter, 1 app/season | Cereals, Southern Europe, autumn-winter, 1 app/season |
| Application doses (g ha^-1^) | 1, 2, 20, 90, 200, 400, 1000, 1200, 15000 | 1, 2, 20, 150, 200, 400, 1000, 1200, 25000 |

* Software available for download (https://esdac.jrc.ec.europa.eu/projects/surface-water)

**Table S3**. Correspondence between Predicted Environmental Concentrations (PECs) of azoxystrobin and tebuconazole in surface water with the respective application rates in the field. Both the EC*x* (calculated for the non-target microalgae used in the mixture assays - *Raphidocelis subcapitata*) and HC*x* (calculated by the SSDs model including all the non-target aquatic organisms) were defined as PECs for the conversion. Recommended and other tested rates of azoxystrobin and tebuconazole were additionally converted to PECs. The specific application rates of each fungicide tested singly or in mixture in the assays with the target fungi, as illustrated in Figure 1, are presented in bold.

|  |  | **Azoxystrobin** | | **Tebuconazole** | |
| --- | --- | --- | --- | --- | --- |
|  |  | **PECs** (ECx/HCx; µg L^-1^) | **Application rates** (g ha^-1^) | **PECs** (ECx/HCx; µg L^-1^) | **Application rates** (g ha^-1^) |
| **Non-target aquatic organisms**  **(SSDs)** | HC_5_ | 30 | 135.6 | 1130 | 5106.2 |
|  | HC_50_ | 390 | 1762.3 | 4710 | 21283.3 |
| ***Raphidocelis subcapitata*** | EC_1_ | 129 | **582.9** | 35 | **158.2** |
|  | EC_5_ | 277 | **1251.7** | 168 | **759.2** |
|  | EC_20_ | 542 | **2449.2** | 672 | **3036.6** |
| **Rates recommended in Custodia®** |  | 20 | **90.0** | 26 | **150.0** |
|  |  | 27 | 120.0 | 35 | 200.0 |
|  |  | 33 | 150.0 | 43 | 250.0 |
| **Other tested rates** |  | 10 | **45** | 13 | **75** |
|  |  | 0 | **0 (control)** | 0 | **0 (control)** |

**Table S4.** Median Effect Concentration (EC_50_) values collected from the literature or estimated herein regarding short-term exposures of aquatic organisms to the fungicides azoxystrobin and tebuconazole. Confidence intervals are shown in brackets, when available.

|  | Species | EC_50_ (mg L^-1^) | Endpoints and timepoints | References |
| --- | --- | --- | --- | --- |
| Azoxystrobin | |  |  |  |
|  | *Anabaena flosaquae* | 9.50 | Biomass 120 h | (EFSA, 2010) |
|  | *Carassius auratus* | 2.71 (2.31-3.04) | Mortality 48h | (Hu et al., 2013) |
|  | *Ctenopharyngodon idella* | 0.34 (0.28-0.41) | Mortality 48 h (juveniles) | (Liu et al., 2013) |
|  | *Daphnia magna* | 0.07 (0.03-0.13) | Mortality 48 h | (Warming et al., 2009) |
|  |  | 0.23 | Mortality 48 h | (EFSA, 2010) |
|  |  | 0.34 (0.32-0.36) | Mortality 48 h | (Ochoa-Acuña et al., 2009) |
|  | *Gammarus fossarum* | 0.15 (0.13-0.17) | Mortality 7 d | (Zubrod et al., 2014) |
|  |  | 0.09 (0.08-0.10) | Feeding rate 7 d | (Zubrod et al., 2014) |
|  | *Gammarus pulex* | 0.27 (0.17-0.45) | Mortality 96 h | (Beketov and Liess, 2008) |
|  | *Lemna gibba* | 2.40 (1.80-3.00) | Yield frond number 7 d | Present study |
|  | *Lemna minor* | 3.05 (1.55-4.56) | Yield frond number 7 d | Present study |
|  | *Lepomis macrochirus* | 1.10 (0.90-1.70) | Mortality 96 h | ECOTOX database* |
|  | *Navicula pelliculosa* | 0.01 | Biomass 120 h | (EFSA, 2010) |
|  |  | 0.15 | Growth rate 120 h | (EFSA, 2010) |
|  | *Oncorhynchus mykiss* | 0.47 (0.40-0.58) | Mortality 96 h | ECOTOX database* |
|  | *Raphidocelis subcapitata* | 0.23 (0.19-0.27) | Growth inhibition 72 h | (Ochoa-Acuña et al., 2009) |
|  | *Thamnocephalus platyurus* | 0.48 (0.41-0.57) | Feeding inhibition 1.5 h | Present study |
|  |  | 0.49 (0.45-0.52) | Mortality 24 h | Present study |
|  | *Xenopus tropicalis* | 0.20 | Mortality 48 h | (Li et al., 2016) |
|  |  | 0.08 | Teratogenic effects 48 h | (Li et al., 2016) |
| Tebuconazole | |  |  |  |
|  | *Brachionus calyciflorus* | 11.59 (10.41-12.77) | Population growth rate 48 h | Present study |
|  | *Chironomus dilutus* | 1.24 (-0.46-2.93) | Immobilization 96 h | (Raby et al., 2019) |
|  | *Cosmarium depressum* | 12.01 | Growth inhibition 96 h | (Da Silveira, 2012) |
|  | *Danio rerio* | 19.70 (18.41-20.80) | Mortality 48-96 h | (Sancho et al., 2010) |
|  | *Daphnia magna* | 11.67 (9.04-14.29) | Feeding inhibition 24 h | Present study |
|  |  | 2.74 (2.33-3.10) | Immobilization 48 h ^1^ | (Qi et al., 2015) |
|  |  | 2.79 | Immobilization 48 h | (EFSA, 2014) |
|  |  | 40.10 | Immobilization 48 h | (Sancho et al., 2009) |
|  |  | 3.53 (3.32-3.78) | Immobilization 48 h ^2^ | (Qi et al., 2015) |
|  | *Desmodesmus communis* | 4.04 | Growth inhibition 96 h | (Da Silveira, 2012) |
|  | *Desmodesmus subspicatus* | 1.96 | Biomass 72 h | (EFSA, 2014) |
|  | *Gammarus pulex* | 1.64 | Mortality 96 h | (Adam et al., 2009) |
|  | *Hyalella azteca* | 1.60 (1.30-1.89) | Immobilization 96 h | (Raby et al., 2019) |
|  | *Lemna minor* | 16.91 (1.84-31.99) | Yield dry weight 7 d | Present study |
|  | *Lepomis macrochirus* | 5.70 | Mortality 96 h | (EFSA, 2014) |
|  | *Leuciscus idus* | 8.70 | Mortality 96 h | (EFSA, 2014) |
|  | *Lumbriculus variegatus* | 6.00 (4.24-8.47) | Imobilization 96 h | (Raby et al., 2019) |
|  | *Neocloeon triangulifer* | 1.03 (0.68-1.37) | Immobilization 96 h | (Raby et al., 2019) |
|  | *Oncorhynchus mykiss* | 4.40 | Mortality 96 h | (EFSA, 2014) |
|  | *Pediastrum boryanum* | 4.13 | Growth inhibition 96 h | (Da Silveira, 2012) |
|  | *Raphidocelis subcapitata* | 2.56 (2.09-3.01) | Yield biomass 72 h | (Coors et al., 2018) |
|  | *Spondylosium pygmaeum* | 3.81 | Growth inhibition 96 h | (Da Silveira, 2012) |
|  | *Thamnocephalus platyurus* | 5.09 (4.56-5.69) | Feeding inhibition 1.5 h | Present study |
|  |  | 10.46 (8.99-11.94) | Mortality 24 h | Present study |
|  | *Xenopus tropicalis* | 2.76 | Teratogenic effects 48 h | (Li et al., 2016) |

*US-EPA ECOTOX database (<https://cfpub.epa.gov/ecotox/>); ^1^S-tebuconazole; ^2^rac-tebuconazole

**Supplementary Material References**

Adam, O., Badot, P.M., Degiorgi, F., Crini, G., 2009. Mixture toxicity assessment of wood preservative pesticides in the freshwater amphipod *Gammarus pulex* (L.). Ecotoxicol. Environ. Saf. 72, 441–449. https://doi.org/10.1016/j.ecoenv.2008.07.017

Beketov, M.A., Liess, M., 2008. Potential of 11 pesticides to initiate downstream drift of stream macroinvertebrates. Arch. Environ. Contam. Toxicol. 55, 247–253. https://doi.org/10.1007/s00244-007-9104-3

Coors, A., Vollmar, P., Sacher, F., Kehrer, A., 2018. Is there synergistic interaction between fungicides inhibiting different enzymes in the ergosterol biosynthesis pathway in toxicity tests with the green alga *Raphidocelis subcapitata*? Ecotoxicology 1–9. https://doi.org/10.1007/s10646-018-1917-5

Da Silveira, S.B., 2012. Toxicidade do tebuconazol em quatro espécies fitoplanctônicas dulcícolas subtropicais. Universidade Federal do Rio Grande - FURG.

EFSA, 2014. Conclusion on the peer review of the pesticide risk assessment of the active substance tebuconazole. EFSA J. 12, 1–98. https://doi.org/10.2903/j.efsa.2014.3485

EFSA, 2010. Conclusion on the peer review of the pesticide risk assessment of the active substance azoxystrobin. EFSA J. 8, 1–110. https://doi.org/10.2903/j.efsa.2010.1542.

Hu, X.G., Liu, L., Hu, K., Yang, X. Le, Wang, G.X., 2013. In Vitro Screening of Fungicidal Chemicals for Antifungal Activity against Saprolegnia. J. World Aquac. Soc. 44, 528–535. https://doi.org/10.1111/jwas.12052

Jonker, M.J., Svendsen, C., Bedaux, J.J.M., Bongers, M., Kammenga, J.E., 2005. Significance testing of synergistic/antagonistic, dose level-dependent, or dose ratio-dependent effects in mixture dose-response analysis. Environ. Toxicol. Chem. 24, 2701–2713. https://doi.org/10.1897/04-431r.1

Li, D., Liu, M., Yang, Y., Shi, H., Zhou, J., He, D., 2016. Strong lethality and teratogenicity of strobilurins on *Xenopus tropicalis* embryos: Basing on ten agricultural fungicides. Environ. Pollut. 208, 868–874. https://doi.org/10.1016/j.envpol.2015.11.010

Liu, L., Jiang, C., Wu, Z.Q., Gong, Y.X., Wang, G.X., 2013. Toxic effects of three strobilurins (trifloxystrobin, azoxystrobin and kresoxim-methyl) on mRNA expression and antioxidant enzymes in grass carp (*Ctenopharyngodon idella*) juveniles. Ecotoxicol. Environ. Saf. 98, 297–302. https://doi.org/10.1016/j.ecoenv.2013.10.011

Ochoa-Acuña, H.G., Bialkowski, W., Yale, G., Hahn, L., 2009. Toxicity of soybean rust fungicides to freshwater algae and *Daphnia magna.* Ecotoxicology 18, 440–6. https://doi.org/10.1007/s10646-009-0298-1

Qi, S.Z., Chen, X.F., Liu, Y., Jiang, J.Z., Wang, C.J., 2015. Comparative toxicity of rac- and S-tebuconazole to *Daphnia magna*. J. Environ. Sci. Heal. - Part B Pestic. Food Contam. Agric. Wastes 50, 456–462. https://doi.org/10.1080/03601234.2015.1018756

Raby, M., Maloney, E., Poirier, D.G., Sibley, P.K., 2019. Acute Effects of Binary Mixtures of Imidacloprid and Tebuconazole on 4 Freshwater Invertebrates. Environ. Toxicol. Chem. 38, 1093–1103. https://doi.org/10.1002/etc.4386

Sancho, E., Villarroel, M.J., Andreu, E., Ferrando, M.D., 2009. Disturbances in energy metabolism of *Daphnia magna* after exposure to tebuconazole. Chemosphere 74, 1171–1178. https://doi.org/10.1016/j.chemosphere.2008.11.076

Sancho, E., Villarroel, M.J., Fernández, C., Andreu, E., Ferrando, M.D., 2010. Short-term exposure to sublethal tebuconazole induces physiological impairment in male zebrafish (*Danio rerio*). Ecotoxicol. Environ. Saf. 73, 370–376. https://doi.org/10.1016/j.ecoenv.2009.09.020

Warming, T.P., Mulderij, G., Christoffersen, K.S., 2009. Clonal Variation in Physiological Responses of *Daphnia Magna* To the. Env. Toxicol Chem 28, 374–380.

Zubrod, J.P., Baudy, P., Schulz, R., Bundschuh, M., 2014. Effects of current-use fungicides and their mixtures on the feeding and survival of the key shredder *Gammarus fossarum*. Aquat. Toxicol. 150, 133–143. https://doi.org/10.1016/j.aquatox.2014.03.002
